# Supplementary material for: The Systems Biology Research Tool: evolvable open-source software
Source: BMC Syst Biol. 2008 Jun 29;2:55. doi: 10.1186/1752-0509-2-55 (PMC2446383; doi:10.1186/1752-0509-2-55)
Supplement: Additional file 1 — SBRT Archive. An archive of the current version of the Systems Biology Research Tool. [file 1752-0509-2-55-S1.zip › sbrt-1.4.0/doc/developers_guide/api/sbrt/shell/text/math/class-use/DirEdgeFormat.html]

Uses of Interface sbrt.shell.text.math.DirEdgeFormat


|  |  |  |
| --- | --- | --- |
| **Overview** \| **Package** \| **Class** \| **Use** \| **Tree** \| **Deprecated** \| **Index** \| **Help** | |  |
| PREV   NEXT | **FRAMES**    **NO FRAMES**     **All Classes** |  |


---


## **Uses of Interface sbrt.shell.text.math.DirEdgeFormat**

| Packages that use DirEdgeFormat | |
| --- | --- |
| **sbrt.shell.mng.graph\_theory** | Provides classes and for creating graph theoretical process managers. |
| **sbrt.shell.text.math** | Provides classes and interfaces for defining formats for mathematical objects. |

Uses of DirEdgeFormat in sbrt.shell.mng.graph\_theory

| Methods in sbrt.shell.mng.graph\_theory with parameters of type DirEdgeFormat | |
| --- | --- |
| `static <N> java.util.Set<DirectedGraph.Edge<N>>` | `PathIdManager.getEdges(java.lang.String fileName, DirEdgeFormat<N> format)`             Parses the file with the provided name, and returns the set of edges it contains. |
| `static <N> DirectedGraph<N>` | `PathIdManager.getGraph(java.lang.String fileName, DirEdgeFormat<N> format)`             Parses the file with the provided name, and returns the directed graph it contains. |

Uses of DirEdgeFormat in sbrt.shell.text.math

| Classes in sbrt.shell.text.math that implement DirEdgeFormat | |
| --- | --- |
| `class` | `DirEdgeFormatV1<N>`             This class is a concrete implementation of `DirEdgeFormat`. |

---


|  |  |  |
| --- | --- | --- |
| **Overview** \| **Package** \| **Class** \| **Use** \| **Tree** \| **Deprecated** \| **Index** \| **Help** | |  |
| PREV   NEXT | **FRAMES**    **NO FRAMES**     **All Classes** |  |


---
